# Cholinergic Signaling Exerts Protective Effects in Models of Sympathetic Hyperactivity-Induced Cardiac Dysfunction

**DOI:** 10.1371/journal.pone.0100179

**Published:** 2014-07-03

**Authors:** Mariana Gavioli, Aline Lara, Pedro W. M. Almeida, Augusto Martins Lima, Denis D. Damasceno, Cibele Rocha-Resende, Marina Ladeira, Rodrigo R. Resende, Patricia M. Martinelli, Marcos Barrouin Melo, Patricia C. Brum, Marco Antonio Peliky Fontes, Robson A. Souza Santos, Marco A. M. Prado, Silvia Guatimosim

**Affiliations:** 1 Department of Physiology and Biophysics, Institute of Biological Sciences, Universidade Federal de Minas Gerais, Belo Horizonte, Brazil; 2 Department of Biochemistry, Institute of Biological Sciences, Universidade Federal de Minas Gerais, Belo Horizonte, Brazil; 3 Department of Morphology, Institute of Biological Sciences, Universidade Federal de Minas Gerais, Belo Horizonte, Brazil; 4 National Institute of Science and Technology in Nanobiopharmaceutics, Universidade Federal de Minas Gerais, Belo Horizonte, MG, Brazil; 5 School of Physical Education and Sport, Universidade de São Paulo, São Paulo, Brazil; 6 Robarts Research Institute, University of Western Ontario, Department of Physiology and Pharmacology, Department of Anatomy & Cell Biology, Schulich School of Medicine & Dentistry, University of Western Ontario, London, Canada; University of Milan, Italy

## Abstract

Cholinergic control of the heart is exerted by two distinct branches; the autonomic component represented by the parasympathetic nervous system, and the recently described non-neuronal cardiomyocyte cholinergic machinery. Previous evidence has shown that reduced cholinergic function leads to deleterious effects on the myocardium. Yet, whether conditions of increased cholinergic signaling can offset the pathological remodeling induced by sympathetic hyperactivity, and its consequences for these two cholinergic axes are unknown. Here, we investigated two models of sympathetic hyperactivity: i) the chronic beta-adrenergic receptor stimulation evoked by isoproterenol (ISO), and ii) the α_2A_/α_2C_-adrenergic receptor knockout (KO) mice that lack pre-synaptic adrenergic receptors. In both models, cholinergic signaling was increased by administration of the cholinesterase inhibitor, pyridostigmine. First, we observed that isoproterenol produces an autonomic imbalance characterized by increased sympathetic and reduced parasympathetic tone. Under this condition transcripts for cholinergic proteins were upregulated in ventricular myocytes, indicating that non-neuronal cholinergic machinery is activated during adrenergic overdrive. Pyridostigmine treatment prevented the effects of ISO on autonomic function and on the ventricular cholinergic machinery, and inhibited cardiac remodeling. α_2A_/α_2C_-KO mice presented reduced ventricular contraction when compared to wild-type mice, and this dysfunction was also reversed by cholinesterase inhibition. Thus, the cardiac parasympathetic system and non-neuronal cardiomyocyte cholinergic machinery are modulated in opposite directions under conditions of increased sympathetic drive or ACh availability. Moreover, our data support the idea that pyridostigmine by restoring ACh availability is beneficial in heart disease.

## Introduction

A substantial amount of data suggests that sympathetic regulation of heart function, via adrenergic receptors, contributes to cardiac remodeling and provides a major pathway for intervention in heart failure (HF) [1], [2], [3]. In contrast, the precise mechanisms required for long-term cholinergic regulation of heart function are only now started to be dissected. Acetylcholine (ACh) is synthesized in the cytoplasm by choline acetyltransferase (ChAT) and stored within vesicles for release. Activity-coupled transport of ACh to synaptic vesicles in nerve-endings is mediated by the vesicular ACh transporter (VAChT) [4], [5]. All ACh released is degraded by cholinesterases mainly, acetylcholinesterase (AChE), to form choline and acetate. Choline is then transported into the cell by the high affinity choline-transporter (CHT1). Cardiac regulation by the parasympathetic nervous system is mediated primarily by ACh binding to the M_2_ muscarinic ACh receptor (M_2_-AChR). Previous studies demonstrated that M_2_-AChR knockout (KO) mice exhibited increased susceptibility to cardiac stress [6], suggesting a protective role for the parasympathetic nervous system in the heart. In line with this finding, vagal stimulation has been shown to be of benefit in heart failure [7]. Mice with reduced expression of the VAChT (VAChT knockdown homozygous mice, VAChT KD^HOM^ mice) [5] have been generated and were pivotal in demonstrating a role for VAChT in sustaining cholinergic tone *in* *vivo*. These mutant mice express only 30% of the normal level of VAChT, and present a number of cholinergic deficits. Our previous publication has shown that chronic decrease of cholinergic tone in VAChT KD^HOM^ mice evoked significant cardiac remodeling characterized by depressed contractile function, calcium (Ca^2+^) signaling dysfunction and altered gene expression in cardiomyocytes [8]. Importantly, these changes were reversed by chronic administration of pyridostigmine, a cholinesterase inhibitor, indicating an important relationship between ACh availability and proper heart function. Altogether, these results support the broad theme that reduced cholinergic function can lead to deleterious effects on the myocardium, and support the notion that increased parasympathetic tone can exert protective effects. In fact, infusion with the cholinesterase inhibitor, neostigmine or with a muscarine receptor agonist, pilocarpine significantly reduced cardiac hypertrophy, reduced TNF alpha levels, elevated interleukin-10 in heart tissues, and improved ventricular function in rats with transverse aortic constriction [9]. Similar cardioprotective effects were observed when another cholinesterase inhibitor, donepezil, was administered to a rat model of myocardial infarction [10] and to a murine model of volume-overloaded congestive heart failure [11]. In addition, previous data from Kanazawa *et al.* [12] have provided evidence that cholinergic transdifferentiation of sympathetic neurons takes place during heart failure and this plays a protective role in the pathogenesis of heart disease. Finally, recent epidemiological data indicate that the use of cholinesterase inhibitors in Alzheimer’s disease associates with 34% reduced risk of myocardial infarction and death [13].

Recently, an intrinsic cardiomyocyte cholinergic system has been described [14], [15], [16] supporting the assumption of local ACh release by ventricular myocytes in the heart. Our group has investigated the importance of this local cholinergic machinery, and presented evidence that ACh released by cardiomyocytes is capable of offsetting the deleterious effects of hyperadrenergic stimulation *in* *vitro* [16]. In addition, removal of VAChT or ChAT exclusively from cardiomyocytes leads to increased levels of oxidative stress and cardiac hypertrophy [17]. Thus, these data bring into perspective another level of cholinergic control of cardiac function that goes beyond the cardiac parasympathetic system, and it is intrinsic to ventricular myocytes. Yet, whether this machinery is regulated *in* *vivo*, and its relationship with parasympathetic activity is unknown.

In this study, our goal was twofold: (i) to assess the consequences of increased adrenergic and cholinergic signaling for the heart and its influence on autonomic function and in the expression levels of cholinergic and adrenergic components in ventricular myocytes, and (ii) to investigate whether activation of cholinergic signaling is capable of offsetting the deleterious effects of increased sympathetic drive *in* *vivo*. Here, we show that upregulation of non-neuronal cholinergic machinery in the heart is observed under conditions of increased sympathetic drive and reduced parasympathetic tone. When ACh availability is increased by pyridostigmine administration cardiac pathological remodeling is prevented and ventricular function is restored in two animal models of sympathetic hyperactivity. Thus, our data indicate that cholinergic signaling might be an important target in heart failure.

## Methods

### Animal models and drug administration

34 Male Wistar rats (250 g) were used in this study. Cardiac hypertrophy was induced by daily intraperitoneal injection of (±) isoproterenol hydrochloride for seven days (4.5 mg/kg body weight) to rats [18]. Isoproterenol was used as a non-specific β-adrenergic agonist (ISO; Sigma, St. Louis, MO, USA). Two distinct doses of Pyridostigmine bromide (0.2 mg/kg body weight or 0.5 mg/kg body weight) were used in this study. The drug was administered twice daily by intraperitoneal injection and treatment started concomitantly with ISO injection. Another group of rats received pyridostigmine alone (0.5 mg/kg body weight). Control animals were saline-injected.

A cohort of 10 male wild-type (control WT group) and 26 congenic α2A/α2C-adrenergic receptor knockout (KO) mice in a C57BL6/J genetic background were studied from 5 to 7 months of age. At this age, these mice display advanced stage cardiomyopathy as previously described [19]. α2A/α2C-KO mice were randomly assigned to receive daily pyridostigmine (3 mg/kg/day) for 28 days. Pyridostigmine was delivered via subcutaneously implanted osmotic mini-pumps.

### Ethics statement

Animals were maintained at the Universidade Federal de Minas Gerais (UFMG), Brazil. This study was approved by the Institutional Animal Care and Use Committee at Universidade Federal de Minas Gerais.

### Vagal and sympathetic function evaluation

The autonomic function and the intrinsic heart rate were evaluated by autonomic blockade using intravenous injections of methylatropine (3 mg/Kg, Sigma) or propranolol (4 mg/Kg, Sigma) at a maximal volume of 0.2 ml per injection after 20 min of baseline conditions. The experiments were performed in two consecutive days. On the first day, methylatropine was the first drug injected and after 15 min propranolol injection was performed. In the second day, to obtain the reverse sequence of the blockade, propranolol was the first drug administered. After 15 min, methylatropine was injected intravenously. The intrinsic HR was evaluated after the injection of methylatropine and propranolol. Using the maximum change of each drug, the sympathetic tone was calculated, on the first day, as the difference between the tachycardia evoked by methylatropine and the intrinsic HR. In the second day, the vagal tone was calculated as the difference between the intrinsic HR and the bradycardic response induced by propranolol [20], [21].

### Echocardiography

Animals were anaesthetized using a nose cone with isoflurane at 5% for one minute and the maintenance dose was 1.25%. The anterior chest was shaved, and the animals were placed in supine position on an imaging stage equipped with built-in electrocardiographic electrodes for continuous heart rate monitoring and a proper heating pad to avoid hypothermia. *In vivo* cardiac function was assessed noninvasively using a high-frequency, high-resolution echocardiographic system consisting of a VEVO 2100 ultrasound machine equipped with a 30–40 MHz bifrequencial transducer (Visual Sonics, Toronto, Canada). High-resolution images were obtained as previously described [22], [23], [24].

### Langendorff-perfused hearts

Briefly, after removal the hearts were perfused with a Krebs-Ringer solution, which was delivered at 37°C in the presence of continuous gassing with 5% CO_2_ to yield a physiological pH of 7.4. Hearts were perfused with this solution for 50 min, as previously described [25], [26].

### Cardiomyocyte isolation and Ca^2+^ recording

Adult ventricular myocytes were freshly isolated and stored in DMEM media (Sigma), until they were used (within 4 h), as previously described [27]. Intracellular Ca^2+^ (Ca^2+^_i_) imaging experiments were performed in Fluo-4 AM (6 µmol/L; Invitrogen, Eugene, OR) loaded-cardiomyocytes for 25 min which were subsequently washed with an extracellular solution that contained 1.8 mmol/L Ca^2+^ to remove the excess dye. Cells were electrically stimulated at 1 Hz to produce steady-state conditions. The confocal line-scan imaging was performed by a Zeiss LSM 510META confocal microscope. For isoproterenol response cardiomyocytes were perfused with Normal Tyrode control solution (in mmol/L, solution 1): NaCl (140), KCl (4), MgCl_2_ (1), CaCl_2_ (1.8), Glucose (10), and HEPES (5); pH = 7.4 adjusted with NaOH. Ca^2+^ transients were first recorded in cardiomyocytes superfused with solution 1, followed by isoproterenol stimulation of cells produced by the addition of 50 nmol/L isoproterenol to solution 1. The myocytes were field-stimulated at 1 Hz and the data plotted as the percentage increase in Ca^2+^ transient fluorescence in the presence of isoproterenol relative to the Ca^2+^ transient obtained in non-stimulated cells.

### Neonatal rat cardiomyocyte culture

Neonatal rat cardiomyocytes were cultured as previously described [28]. Briefly, cardiac cells were plated in dishes containing M199 medium supplemented with 100 units/mL penicillin, 100 µg/mL streptomycin, 10% Fetal Bovine Serum and 2 mmol/L L-glutamine. To prevent growth of fibroblasts, medium was supplemented with 20 µg/mL cytosine-d-arabinofuranoside (ARA-c) for 48 hours. For siRNA studies, neonatal cardiomyocyte cultures at day 4 were transfected with 100 nM of siRNA. The cells were incubated at 37°C in an atmosphere of 5% CO_2_ for 30 h and then exposed to isoproterenol (10 µmol/L) for another 24 h prior to use. The cells were then used for qPCR analyses. The siRNA for AChE was prepared as previously described [16].

### Quantitative real time PCR

For RNA purifications, tissues were grounded in a potter and pestle with liquid nitrogen and total RNA was extracted using TRIzol® (Invitrogen). For quantitative PCR (qPCR), total RNA was treated with DNase I (Ambion, Austin, TX, USA) and first strand cDNA was synthesized using High Capacity cDNA Transcription Kit (Applied Biosystems, CA, USA) according to the manufacturer’s instructions. After reverse transcription, the cDNA was subjected to qPCR on a 7500 Real Time PCR System (Applied Biosystems, CA, USA) using Power SYBR Green PCR Master Mix (Applied Biosystems, CA, USA), as previously described [8].

### Cardiac morphometry

Heart hypertrophy was evaluated by morphometry (n = 5 per group). The rats were anesthetized with 10% ketamine/2% xylazine (4∶3, 0.1 mL/100 g, i.p.) and heart beat was stopped in diastole using 10% KCl (i.v.). Hearts were immersed in 4% paraformaldehyde in 0.1 M phosphate buffer, pH 7.4 for 24 hours at room temperature. The tissues were dehydrated by sequential washes with 70, 80, 90 and 100% ethanol and embedded in paraffin. Transversal sections (5 µm) were cut starting from base area of the heart at intervals of 40 µm and stained with hematoxylin-eosin for cell morphometry. Tissue sections (2 for each animal) were examined with Axioplan 2 Zeiss microscope, and analyzed with ImageJ software. Only digitized images of cardiomyocytes cut longitudinally with nuclei and cellular limits visible were used for analysis (an average of 40 cardiomyocytes for each animal). The diameter of each myocyte was measured across the region corresponding to the nucleus.

### Western blot

40–60 µg of protein were separated by SDS-PAGE 10% gels. Antibodies and their sources are as follows: anti-SERCA2 (1∶1000 ABR), and anti-GAPDH (1∶4000 Santa Cruz Biotechnology). Immunodetection was carried out using enhanced chemiluminescence (Amersham Biosciences). Protein levels were expressed as a ratio of optical densities. GAPDH was used as a control for any variations in protein loading.

### Statistical analysis

All data are expressed as means ± SEM, and the number of cells or experiments is shown as *n*. Significant differences between groups were determined with a Student's *t*-test or ANOVA followed by the Newman-Keuls *post hoc* test. Values of *p*<0.05 were considered to be statistically significant.

## Results

**Figure 1:**
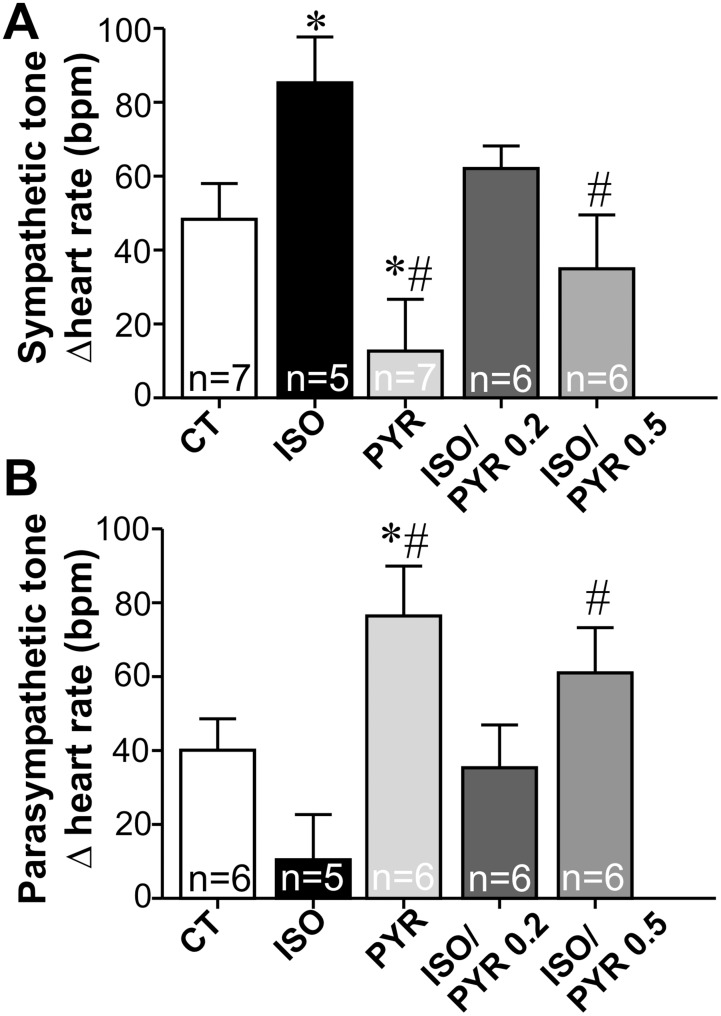
Cholinesterase inhibition alters the activity of the cardiac autonomic system evoked by isoproterenol. Changes in heart rate (HR) under the inhibition of parasympathetic and/or sympathetic nervous system were monitored in rats injected with methylatropine and propranolol for further calculation of sympathetic and vagal tone, as described in Methods. Two different doses of pyridostigmine were combined with isoproterenol in this study (0.2 mg/kg body weight or 0.5 mg/kg body weight). **A–B.** ISO administration to rats for seven days resulted in increased sympathetic tone when compared with control group. Vagal tone was lower in isoproterenol treated rats when compared to control rats. The higher dose of pyridostigmine was more efficient in preventing the isoproterenol-induced changes in heart rate. Rats treated with pyridostigmine alone (0.5 mg/kg body weight) presented reduced sympathetic and increased parasympathetic tone, respectively. n = number of rats. *p<0.05 when compared to control group and #p<0.05 when compared to the ISO-treated rat using Student's *t* test.

Cholinergic signaling in the heart depends on the parasympathetic nervous system as well as on the recently described cardiomyocyte cholinergic machinery [14], [15], [16]. Firstly, we assessed whether cholinergic activity at the parasympathetic nerve endings was modulated in rats with increased sympathetic drive. To assess the activity of the cardiac autonomic nervous system, we monitored changes in heart rate (HR) under the inhibition of parasympathetic and/or sympathetic nervous tone in control and isoproterenol (ISO)-treated rats. Fig. 1 shows HR responses to intravenous injections of methylatropine and propranolol used to calculate the cardiac sympathetic and vagal tone. As shown in Fig. 1A, ISO administration to rats for seven days produces an autonomic imbalance characterized by increased cardiac sympathetic tone when compared with control group (p<0.05). In contrast, cardiac vagal tone tended to be lower in ISO rats when compared to control (Fig. 1B). When cholinergic signaling was increased by administration of the cholinesterase inhibitor pyridostigmine to rats opposite results were observed. Pyridostigmine-treated rats displayed reduced sympathetic and increased parasympathetic tone, respectively. In order to assess the consequences of increased cholinergic function to the diseased heart we treated rats simultaneously with ISO and pyridostigmine. Two distinct doses of pyridostigmine were used in this study (0.2 mg/kg body weight or 0.5 mg/kg body weight), as shown in Fig. 1A–B. Pyridostigmine administration to ISO rats prevented changes in parasympathetic and sympathetic tone, with significant results observed at the higher dose. Altogether, our data show that both ISO and pyridostigmine are capable of modulating cardiac sympathetic and parasympathetic tone in opposite ways, and that increased ACh availability induced by pyridostigmine administration to rats is capable of overcoming isoproterenol induced changes in autonomic function.

**Figure 2:**
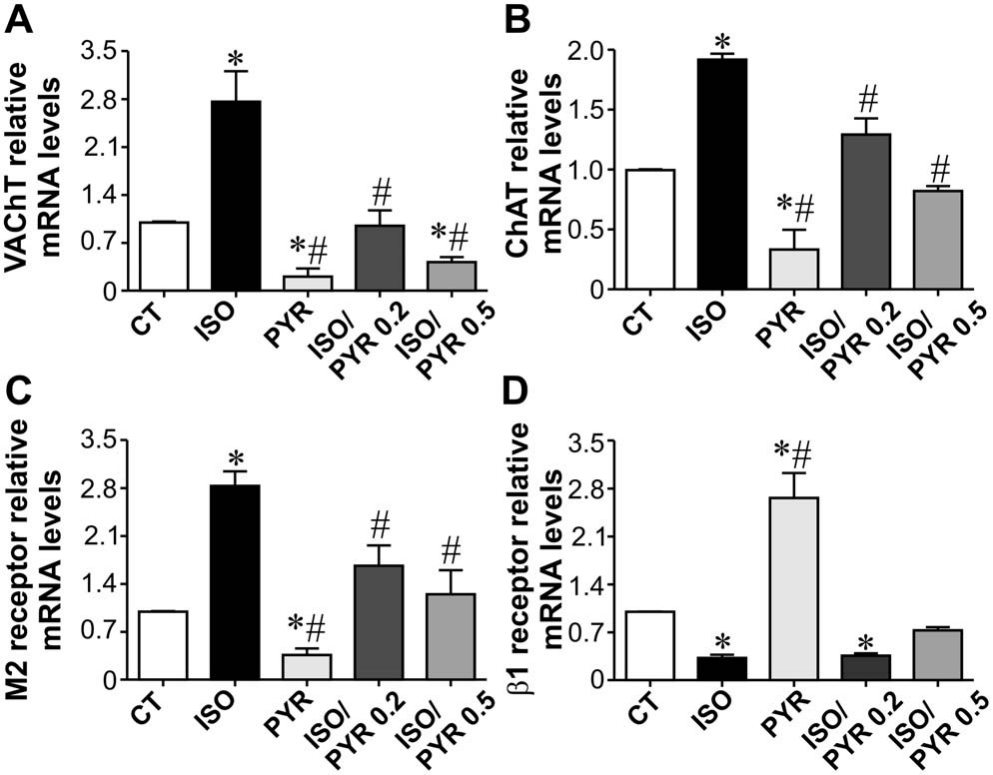
Opposite modulation of cardiomyocyte cholinergic machinery by isoproterenol and pyridostigmine. **A–C**. qPCR experiments show that cardiomyocytes from ISO-rats present upregulation of VAChT, ChAT and M_2_ muscarinic receptor mRNA levels. Cholinesterase inhibition prevented these effects. Lower levels of VAChT, ChAT and M_2_ ACh muscarinic receptor transcripts were observed in cardiomyocytes from pyridostigmine treated rats. **D.** Downregulation of β1-AR transcript is observed in ventricular myocytes from ISO-rats, while pyridostigmine-treated rats presented opposite results. Cholinesterase therapy partially reversed the effect of ISO on β1-AR transcripts. n = 4–6 cardiomyocyte preparations from each group. *p<0.05 when compared to control group and #p<0.05 when compared to the ISO-treated rat.

To investigate whether the non-neuronal cardiomyocyte cholinergic machinery is modulated *in* *vivo* in ISO rats, we isolated ventricular myocytes and investigated expression levels of cholinergic components. mRNA levels of VAChT, ChAT and M_2_ muscarinic receptors were upregulated in cardiac cells isolated from ISO-treated rats (Fig. 2A–C). In contrast, lower levels of VAChT, ChAT and M_2_ mRNA were found in rats with increased ACh availability due to cholinesterase inhibition. As shown in Fig. 2A–C, both doses of pyridostigmine used in this study were efficient in preventing the upregulation of the cholinergic machinery induced by isoproterenol. Taken together, these data indicate that reprogramming of cardiomyocyte cholinergic machinery is under opposing modulation by isoproterenol and pyridostigmine. Moreover, our finding that cardiomyocyte cholinergic proteins are modulated *in* *vivo* is potentially important since it suggests that this intrinsic ACh synthesis site can be activated directly in the ventricles under hyperadrenergic conditions, and this occurs in parallel with a reduction in parasympathetic tone. In order to extend these findings and to better understand under what conditions this intrinsic cholinergic system is modulated, we next performed studies in isolated cardiac cells and assessed the expression levels of VAChT mRNA in response to increased adrenergic and cholinergic stimulation. In order to deplete endogenous acetylcholinesterase and simulate conditions of increased cholinergic signaling in rat cardiomyocytes, we used specific siRNA targeting AChE. In rat cardiomyocytes, VAChT mRNA was upregulated by isoproterenol, while cells treated with an AChE-siRNA presented significantly decreased VAChT mRNA levels (Fig.S1). Importantly, AChE silencing partially abolished the effect of isoproterenol on VAChT expression levels. Thus these data further support our *in* *vivo* findings and confirm the hypothesis that non-neuronal cholinergic signaling is under differential modulation by adrenergic and cholinergic signals.

We also assessed expression levels of β1-adrenergic receptor in ventricular myocytes from ISO and pyridostigmine-treated rats. As shown in Fig. 2D, 7-day ISO injection to rats led to a significant β1-adrenergic receptor downregulation. In contrast, ventricular myocytes from pyridostigmine treated rats presented a robust increase in β1-AR transcript levels. Administration of pyridostigmine to ISO-rats attenuated the ISO-induced-β1-AR downregulation. Collectively, our data from Fig. 2 suggest that cholinergic signaling is activated in ventricular myocytes under conditions of increased adrenergic drive, while expression of β1 receptors is downregulated. Interestingly, both effects were prevented when ACh availability was increased upon pyridostigmine treatment.

**Figure 3:**
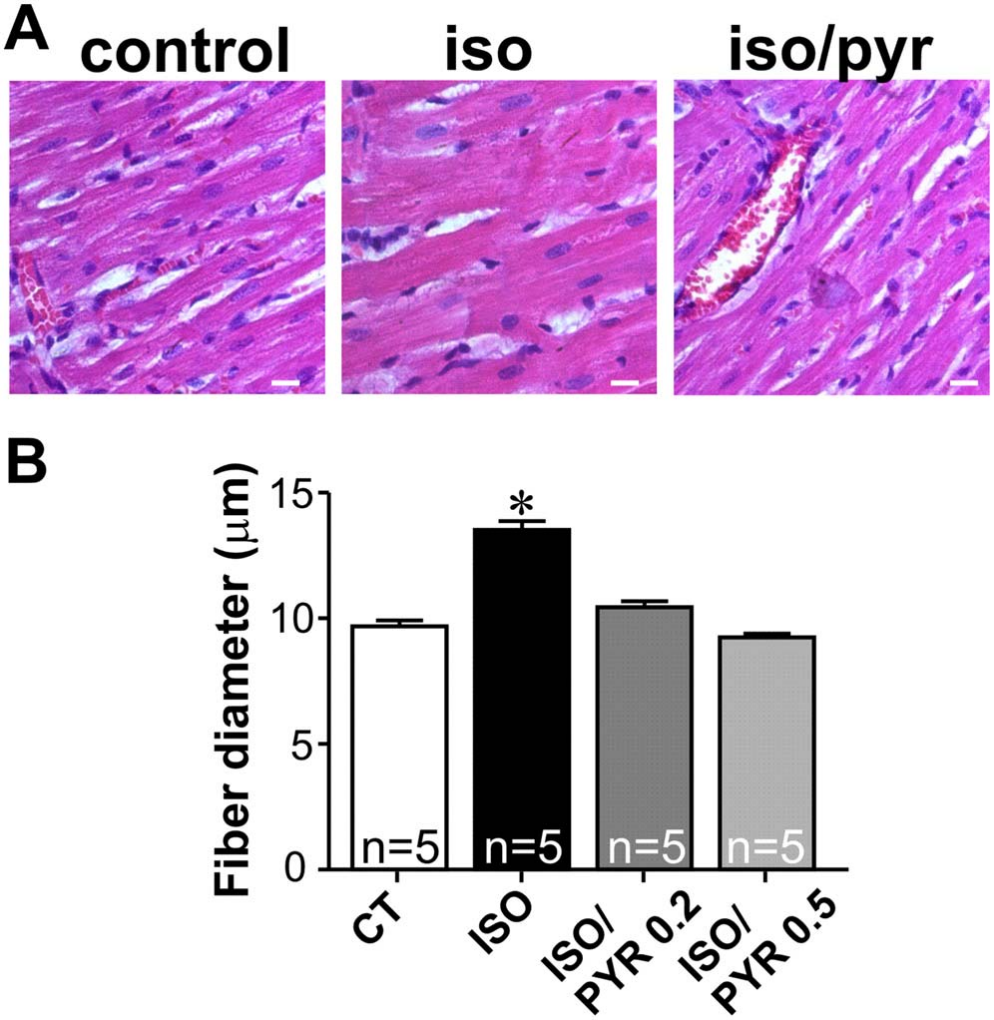
Cholinesterase inhibition prevents isoproterenol induced-hypertrophy. **A.** Representative images. **B.** Morphometry analyses showing the increase in fiber diameter in rats subjected to seven day ISO administration. Cholinesterase inhibition prevented the ISO-induced cardiomyocyte hyperthophy. n = number of hearts analysed. *p<0.05 when compared to other groups. Scale bar = 10 µm.

**Figure 4:**
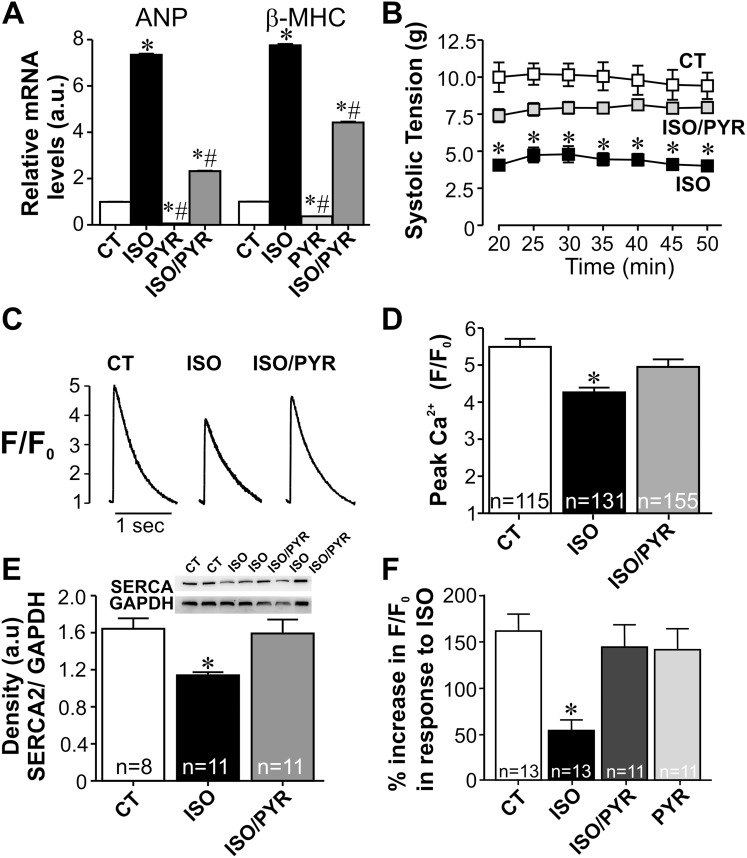
Increased cholinergic signaling functionally prevents ISO-induced pathological remodeling. **A.** Differential effects of isoproterenol and pyridostigmine on transcripts for ANP and β-MHC were detected by real-time PCR in cardiomyocytes from each experimental group. Administration of pyridostigmine prevents the upregulation of hypertrophic markers in cardiomyocytes from ISO-rats. n = 4–6 cardiomyocyte preparations from each group. *p<0.05 when compared to control group and #p<0.05 when compared to the ISO-treated rat. **B.** Pyridostigmine treatment restored systolic tension in ISO-treated rats back to control levels. n = 7 hearts per group. **C.** Representative profile of the Ca^2+^ transient. **D.** Bar graph shows averaged-peak Ca^2+^ transient. Significant reduction in peak Ca^2+^ transient is observed in cardiomyocytes from ISO-rats. Anticholinesterase therapy significantly prevented intracellular Ca^2+^ dysfunction in cardiomyocytes from ISO-rats. n = number of ventricular myocytes analysed. **E.** Top, representative western-blot. Bottom, averaged-densitometry. Pyridostigmine-treatment prevents SERCA2 downregulation in ISO cardiomyocytes. **F.** % Increase in cytosolic Ca^2+^ concentration ([Ca^2+^]_i_) above basal level measured 2 minutes after stimulation with 50nmol/L isoproterenol. β-adrenergic stimulation increased Ca^2+^ transient amplitude in cardiomyocyters from saline-treated group more than in cells from ISO-rats. Cardiomyocytes from ISO/PYR rats presented a similar increase in [Ca^2+^]_i_ transient amplitude in response to acute ISO perfusion as control cells. * = p<0.05 when compared to other experimental groups. n = number of cells analysed from 4 different animals in each group.

To investigate the functional consequences of these changes in cholinergic signaling for the heart, we performed morphometric and echocardiographic analyses. Morphometric analyses of fiber diameter revealed a significant increase in cardiomyocyte hypertrophy in ISO rats (Fig. 3A–B). Pyridostigmine alone, had no effect on cardiomyocyte size (data not shown), but when combined with isoproterenol the drug was capable of preventing ISO-induced hypertrophy. Since the higher pyridostigmine dose was more effective in preventing autonomic imbalance and cardiomyocyte hypertrophy, we performed all the remaining experiments in ISO rats treated with the higher dose of pyridostigmine. Real-time experiments supported the morphometry data since transcript levels of hypertrophic markers atrial natriuretic peptide (ANP) and β-myosin heavy chain (β-MHC) were upregulated in cardiomyocytes from ISO-rats (Fig. 4A), an effect that was attenuated in cardiomyocytes from ISO-rats treated concomitantly with pyridostigmine. Interestingly, pyridostigmine treatment alone significantly decreased fetal gene expression when compared to control. Table 1 shows that after seven days of ISO administration to rats there was a significant increase in ejection fraction (EF) and fractional shortening (FS) indicating that at this stage ventricular function was still preserved. Interestingly, concomitant treatment with pyridostigmine produced a similar increase in EF and FS. In spite of enhanced ejection fraction, pronounced cardiac remodeling was observed in ISO-rats. As shown in Table 1, ISO injection led to significant thickening of the left ventricular posterior wall and septum both in systole and diastole with most of these changes significantly attenuated by pyridostigmine. In addition, the left ventricular internal dimension at diastole was significantly reduced in ISO-rats. This alteration was accompanied by a decrease in end-diastolic volume. As shown in Table 1, both changes were prevented by pyridostigmine. Except for an increase in chamber dimension at diastole, pyridostigmine treatment had no effect on cardiac parameters. Thus, these functional data clearly indicate that increased cholinergic signaling induced by cholinesterase inhibition significantly attenuated isoproterenol-induced pathological remodeling.

**Table 1:** Echocardiographic parameters.

Parameter	CT (n = 8)	ISO (n = 8)	ISO+PYR (n = 9)	PYR (n = 4)
Cardiac output (mL/min)	65.09±3.03	48.74±6.11*	71.22±2.31&	76.64±3.84&
LV internal dimension (diastole,mm)	6.87±0.18	5.92±0.18*	6.83±0.15&	7.63±0.22*& #
LV internal dimension (systole,mm)	4.08±0.10	3.24±0.20*	3.48±±0.13*	4.28±0.39&#
LV posterior wall (systole, mm)	2.29±0.08	3.27±0.19*	2.85±0.15*	2.60±0.17&
LV posterior wall (diastole, mm)	1.43±0.05	2.19±0.14*	1.81±0.10&	1.54±0.17&
interventricular septal dimension(systole, mm)	1.90±0.10	2.67±0.13*	2.36±0.07*&	1.90±0.13&#
interventricular septal dimension(diastole, mm)	1.05±0.04	1.57±0.07*	1.29±0.04*&	1.18±0.06&
LV ejection fraction (%)	72.21±1.74	78.94±1.28*	79.1±1.73*	73.72±3.32
LV fractional shortening (%)	41.3±1.73	47.5±1.31*	51.0±1.57*	44.34±2.97
LV systolic volume (µL)	178.2±8.98	134.2±15.92*	200.0±5.33&	215.8±13.73&
End diastolic LV volume (µL)	246.2±9.12	169.9±19.35*	253.0±4.85&	293.8±19.06&
End systolic LV volume (µL)	68.04±4.37	35.63±4.15*	52.98±4.58*&	77.97±11.69&#
Heart rate (bpm)	367.0±11.11	361.5±5.79	356.4±8.61	356.1±5.56

Echocardiographic measurements of cardiac parameters in rats following isoproterenol injection for 7 days. Pyridostigmine treatment of ISO-rats attenuated the development of cardiac remodeling induced by isoproterenol. n = number of rats analysed in each experimental group.

* = p<0.05 versus CT,

& = p<0.05 versus ISO,

# = p<0.05 versus ISO+PYR.

To further assess the consequences of cholinergic signaling activation by pyridostigmine in the diseased heart we used the Langendorff-isolated heart preparation. As shown in Fig. 4B, a marked decrease in systolic tension was observed in hearts from the ISO group. This finding contrasted with echocardiography data where heart function was increased, indicating that *in* *vivo* compensatory mechanisms were contributing to preserve cardiac function in ISO rats. Combined administration of ISO and pyridostigmine resulted in significant improvement of cardiac systolic tension when compared to hearts from ISO-rats. Pyridostigmine, alone had no effect on systolic tension (data not shown). To further investigate the underlying cellular basis for the effects of pyridostigmine on ISO-injected rats, we examined intracellular Ca^2+^ in freshly isolated Fluo-4AM loaded ventricular myocytes. Figure 4C shows representative profiles of intracellular Ca^2+^ transient recorded in Fluo-4AM loaded ventricular myocytes. As shown in Figure 4D, ventricular myocytes from ISO rats present a significant reduction of the peak calcium transient (p<0.05). The impaired Ca^2+^ handling observed in cells from ISO rats corroborates data obtained by the Langendorff technique. In contrast, the amplitude of the [Ca^2+^]_i_ transient in cardiomyocytes from rats treated with a combination of pyridostigmine and ISO was not statistically different from that obtained in control cardiomyocytes. The reduced amplitude of the [Ca^2+^]_i_ transient in ISO-cardiomyocytes is consistent with the decreased expression of the sarcoplasmic reticulum Ca^2+^ pump (SERCA2), therefore we examined SERCA2 levels in these hearts. As shown in Fig. 4E, SERCA2 expression levels were significantly reduced in cardiomyocytes from ISO-treated rats relative to controls. In contrast, cardiomyocytes from rats treated with a combination of pyridostigmine and ISO, presented normal levels of SERCA2.

Since pyridostigmine administration prevented the ISO-induced β-1 receptor downregulation, we addressed the functionality of these receptors by assessing the percentage increase in Ca^2+^ transient amplitude upon ISO stimulation. Upon reaching steady state in our normal Tyrode control solution, perfusion of myocytes with a sub-maximal dose of ISO (50 nmol/L) produced a large increase in Ca^2+^ transient amplitude in cells from control rats. This effect is shown in Fig. 4F, which shows the % Δ in response to ISO. As expected, perfusion with ISO had a less pronounced effect on the Ca^2+^ transient amplitude in myocytes from ISO-rats, indicating impaired β-adrenergic responsiveness of cells from this group. In contrast, cardiomyocytes from rats treated with a combination of ISO and pyridostigmine presented normal response to ISO stimulation, comparable to that observed in cells from control rats. Thus, pyridostigmine treatment restores the functionality of β-adrenergic receptors in ISO-myocytes corroborating our real time PCR data. Taking together, our data show that increased cholinergic signaling induced by pyridostigmine is capable of preventing deleterious effects of increased sympathetic drive.

**Figure 5:**
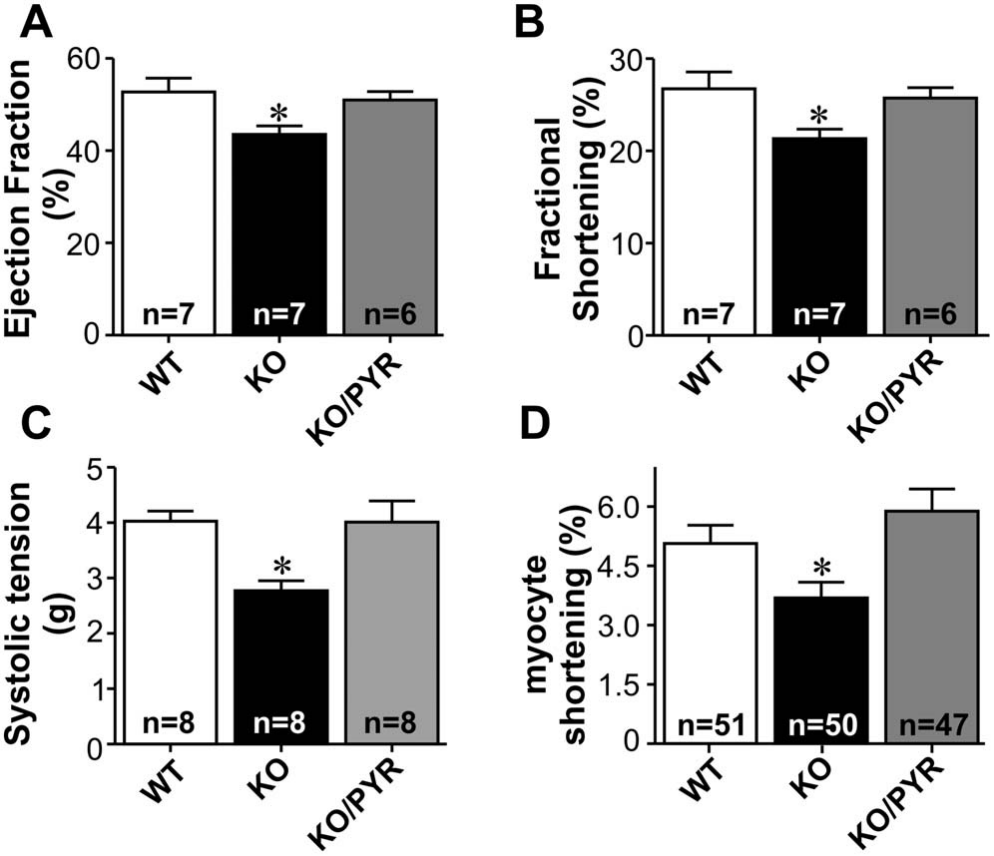
Increased cholinergic signaling improves cardiac function in mice with sympathetic hyperactivity-induced heart failure. **A–B**. Percentage increase in left ventricular ejection fraction and fractional shortening measured by echocardiography. Systolic tension (**C**) and cardiomyocyte shortening (**D**) measurements were performed in WT and α_2A_/α_2C_-KO mice treated or not with pyridostigmine. Cholinesterase inhibition therapy restored cardiac function and and cellular contractile function of α_2A_/α_2C–_KO mice to control levels. *p<0.05 when compared to WT and α_2A_/α_2C–_KO/PYR.

To investigate whether increased cholinergic signaling would also revert pre-existing sympathetic hyperactivity-induced heart failure, we treated α_2A_/α_2C_-KO mice with pyridostigmine. Mice lacking both α_2A_-and α_2C_- adrenergic receptors (α_2A_/α_2C_-KO) develop sympathetic hyperactivity-induced heart failure [19], [29]. The first signs of heart failure are observed at age of 5 months. Fig. 5A–B shows that α_2A_/α_2C_-KO mice present reduced left ventricular ejection fraction and fractional shortening as assessed by echocardiography, and administration of pyridostigmine for 28 days significantly increased these parameters towards control levels. These data confirm our previous finding that baseline fractional shortening was significantly reduced in α_2A_/α_2C_-KO mice, when compared with control mice [19]. To further assess directly the heart dysfunction in α_2A_/α_2C–_KO mice we used the Langendorff-isolated heart preparation. In agreement with the results obtained with echocardiography studies, isolated hearts from α_2A_/α_2C–_KO mice had significantly decreased systolic tension when compared to WT hearts (Fig. 5C). Pyridostigmine treatment reversed the decreased systolic tension observed in α_2A_/α_2C–_KO hearts. Similarly, a contractile dysfunction was observed in freshly isolated adult ventricular myocytes from α_2A_/α_2C_-KO mice, which demonstrated reduced fractional shortening when compared to cardiomyocytes from WT mice (Fig. 5D). Cholinesterase inhibition of α_2A_/α_2C_-KO mice reversed this change thus confirming that increased cholinergic signaling protects against chronic adrenergic stimulation.

## Discussion

Here, we show that both cholinergic axes, the cardiac parasympathetic system and non-neuronal cardiomyocyte cholinergic machinery are modulated in opposite directions under conditions of increased sympathetic drive or of increased ACh availability. As such, in a model of increased adrenergic stimulation, parasympathetic tone is reduced while a significant induction of VAChT, ChAT and M_2_-receptors is observed in ventricular myocytes, thus uncovering a new site of ACh synthesis in the ventricles *in* *vivo*. When ACh availability increases, for example by administration of pyridostigmine, this effect is suppressed. In the presence of pyridostigmine, normal parasympathetic response is restored and pathological remodeling induced by increased sympathetic drive is prevented. Thus, our findings indicate that therapies aimed at enhancing cholinergic activity might be an important target in heart failure.

### Modulation of cardiomyocyte cholinergic machinery *in* *vivo*

We have previously shown that ventricular myocytes express cholinergic components ChAT, VAChT, CHT1 and muscarinic receptor type 2 and release ACh [16], [17]. Here, we show that this intrinsic cholinergic machinery is upregulated *in* *vivo* and *in* *vitro* by isoproterenol, suggesting that under this condition ACh release by cardiomyocytes is increased. These data corroborate our previous finding that isoproterenol treatment *in* *vitro* leads to a significant increase in expression levels of cholinergic components in neonatal cardiomyocytes [16]. A possibility raised by these findings is that ACh released by cardiomyocytes acts as a feedback signal to counteract excessive adrenergic stimulation thus exerting protective effects. Evidence supporting this hypothesis comes from the fact that when ACh synthesis by cardiomyocytes is blocked, for example in vesamicol treated myocytes isoproterenol treatment leads to a greater increase in cell surface area when compared with cells treated with isoproterenol alone [16]. Therefore, in the absence of VAChT the extent of cardiac hypertrophy induced by isoproterenol is increased corroborating the fact that cardiomyocyte cholinergic machinery exerts protective effects. In addition, mice with targeted deletion of VAChT in cardiomyocytes present cardiac dysfunction, increased ROS levels, and cardiomyocyte hypertrophy [17]. Similar findings were also observed in another genetic mouse model with ablation of ChAT (the enzyme responsible for ACh synthesis) in cardiomyocytes [17]. Along these lines, Kakinuma *et al*. have demonstrated that increased expression of ChAT in cardiomyocytes turns the heart more resistant to injury caused by ischemia and reperfusion [30]. Taking all these into consideration, it appears that under stress conditions the cardiomyocyte cholinergic machinery is activated and this may contribute to cardiac protection. When ACh availability is restored, for example during cholinesterase therapy, upregulation of cardiomyocyte cholinergic machinery is no longer required. This assumption is further supported by our finding that pyridostigmine administration *in* *vivo* leads to a significant reduction in VAChT, ChAT, and M_2_ expression, and by our *in* *vitro* data showing that cardiomyocytes transfected with AChE siRNA present significant reduction in VAChT mRNA. It is worth noting the fact that this cardiomyocyte intrinsic cholinergic machinery is subjected to opposing modulation by isoproterenol and pyridostigmine, suggesting that cardiac cells may represent another level of cholinergic control of heart function, that goes beyond the parasympathetic autonomic system.

### Cardioprotective effects of cholinesterase inhibition

The present results with pyridostigmine are consistent with the idea that enhancing ACh signaling may limit adverse remodeling and cardiac deterioration by controlling cardiac gene expression, preventing cardiomyocyte hypertrophy, and therefore preserving Ca^2+^ cycling and contractile function. Taken together, these results provide the cellular basis to attempt to improve cholinergic neurotransmission and restore parasympathetic-sympathetic balance to influence the outcome of heart failure in clinical settings.

Previous data in the literature have shown that treatment with pyridostigmine improves cardiocirculatory parameters in rats with chronic heart failure due to coronary artery ligation [31], [32]. Here, we extended these findings by showing the mechanisms involved in the protective effects of cholinesterase inhibition therapy. Accordingly, pyridostigmine improves cardiac function by preventing the increase in sympathetic activity, hypertrophic growth, and by improving calcium dynamics. The signaling pathways mediating these effects, however, have yet to be elucidated. In addition, we found that pyridostigmine can reverse pre-existing pathological remodeling in a model of hyperadrenergic induced heart failure, indicating that increased ACh availability is associated to cardioprotection. Overall, these findings are consistent with a recent study using a cohort of 7073 subjects showing that the use of cholinesterase inhibitor was associated with a reduced risk of myocardial infarction and death [13].

Importantly, beneficial effects of cholinesterase inhibition are not restricted to the synaptic cleft but also observed locally in ventricular myocytes. Ventricular myocytes are known to express AChE [16], and its inhibition leads to a local increase in cardiomyocyte derived-ACh [17]. In cardiac cells, ACh acting through M_2_ receptors activates nitric oxide synthesis exerting anti-hypertrophic effects [16]. In addition, we have demonstrated that neonatal rat cardiomyocytes transfected with siRNA directed to AChE presented reduced expression levels of ANP and β-MHC, and were protected from hypertrophic effects of isoproterenol [16].

Taking together, our data suggest that the cardiomyocyte cholinergic machinery plays a role as an adaptative defense mechanism activated under conditions of increased sympathetic drive. Increased cholinergic tone induced by pyridostigmine is important to restore the balance of the autonomic nervous system and prevent cardiovascular dysfunction in two models of sympathetic hyperactivity. Overall our data support the fact that therapies aimed at enhancing cholinergic signaling in the synaptic cleft and locally in the heart might be an important target in heart failure.

## Supporting Information

Figure S1 **Adrenergic and cholinergic signals regulate VAChT mRNA levels in neonatal cardiomyocytes.** siRNA targeting AChE significantly reduces mRNA expression levels of VAChT. Isoproterenol treatment significantly increases VAChT mRNA levels in neonatal cardiomyocytes, an effect that is significantly attenuated in cells transfected with siRNA targeting AChE. n = 4 samples from each group. *p<0.05 when compared to control group and #p<0.05 when compared to the ISO-treated cardiomyocytes.
(TIF)
