# Supplementary figures and images for: Cholinergic Signaling Exerts Protective Effects in Models of Sympathetic Hyperactivity-Induced Cardiac Dysfunction
Source: PLoS One. 2014 Jul 3;9(7):e100179. doi: 10.1371/journal.pone.0100179 (PMC4081111; doi:10.1371/journal.pone.0100179)

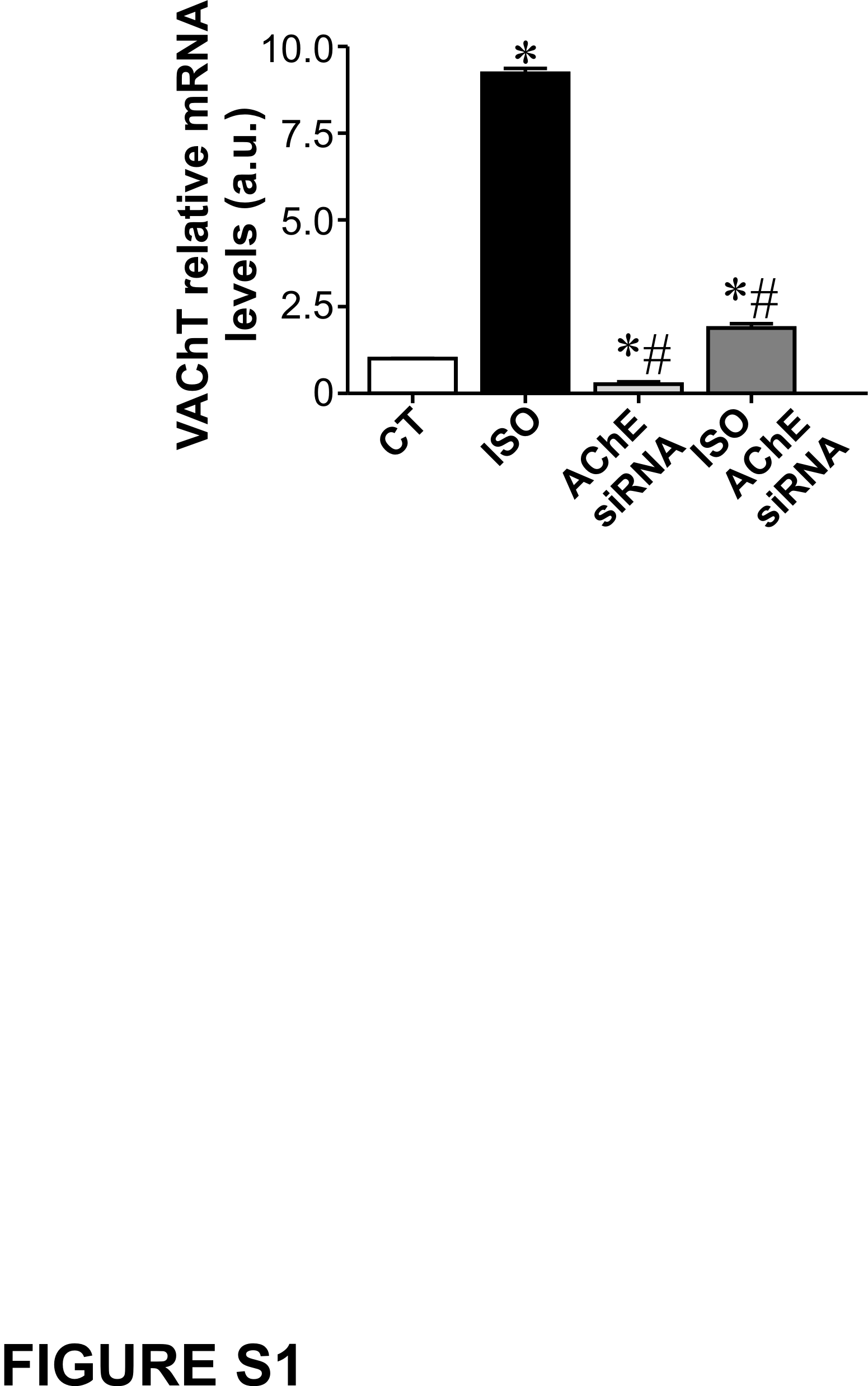

Supplement: Figure S1 — Adrenergic and cholinergic signals regulate VAChT mRNA levels in neonatal cardiomyocytes. siRNA targeting AChE significantly reduces mRNA expression levels of VAChT. Isoproterenol treatment significantly increases VAChT mRNA levels in neonatal cardiomyocytes, an effect that is significantly attenuated in cells transfected with siRNA targeting AChE. n = 4 samples from each group. *p<0.05 when compared to control group and #p<0.05 when compared to the ISO-treated cardiomyocytes. (TIF) [file pone.0100179.s001.tif]
